# Synthesis, Characterization, and Antifogging Application of Polymer/Al_2_O_3_ Nanocomposite Hydrogels with High Strength and Self-Healing Capacity

**DOI:** 10.3390/polym10121362

**Published:** 2018-12-08

**Authors:** Bo Xu, Yuwei Liu, Jiugang Yuan, Ping Wang, Qiang Wang

**Affiliations:** Key Laboratory of Eco-Textile, Ministry of Education, College of Textile and Clothing, Jiangnan University, Wuxi 214122, China; boxu@jiangnan.edu.cn (B.X.); 18861822651@163.com (Y.L.); jiugangyuan@jiangnan.edu.cn (J.Y.); wxwping@163.com (P.W.)

**Keywords:** nanocomposite hydrogels, Al_2_O_3_, high-strength, self-healing, antifogging

## Abstract

Hydrogels with outstanding mechanical performance, self-healing capacity, and special functionality are highly desirable for their practical applications. However, it remains a great challenge to achieve such hydrogels by a facile approach. Here, we report a new type of nanocomposite hydrogels by in situ copolymerization of acrylic acid (AA) and 2-acrylamido-2-methylpropane sulfonic acid (AMPS) using alumina nanoparticles (Al_2_O_3_ NPs) as the cross-linkers. The obtained hydrogels are highly stretchable and compressible, which could sustain large-scale extension (>1700%) or compression (90%) without failure, and exhibit tensile and compressive strength up to 660 kPa and 8.3 MPa, respectively. Furthermore, this kind of hydrogel also display considerable self-healing capacity due to their noncovalent cross-linking mechanism, as well as the hydrogen-bonding interactions between polymer chains. More interestingly, it was found that the resultant gels possess a long-lasting antifogging property that could prevent the formation of fog on the glass plate above hot water for at least 90 min. It is expected that this novel type of hydrogel would show great promise for various applications, including soft robots, artificial muscles, and optical devices.

## 1. Introduction

Polymer hydrogels have attracted considerable research interest as advanced biomaterials because of their unique combination of solidlike appearance, high hydrophilicity, excellent biocompatibility, and tunable mechanical properties, as well as their responsiveness to environmental stimuli [1,2,3,4,5]. Therefore, hydrogels have been extensively explored for applications including biomedicine [6,7], tissue engineering [8,9], drug delivery [10,11], biosensing [12,13], and wound dressing [14,15,16]. However, the intrinsic mechanical weakness of conventional chemically cross-linked hydrogels caused by their random distribution of cross-linking points or/and the lack of an energy-dissipation mechanism severely restricted their vast applications in load-bearing systems such as soft machines [17,18], bioactuators [19], and artificial tissue [20,21]. To conquer this limitation, numerous synthetic strategies have been attempted to improve the mechanical properties of hydrogels, and a series of tough and robust hydrogels have been realized [22]. For instance, Ito et al. reported a topological hydrogel (TP gel) with “figure-of-eight” cross-links. When the gel is stretched, the cross-linking points could slide along the polymer chains, which evenly distribute the external loads within the polymer matrix [23,24]. Gong et al. prepared a type of double-network hydrogels (DN gels) with two interpenetrated cross-linking networks with different cross-linking densities [25,26]. Upon loading, the densely cross-linked network factures to dissipate energy, while the loosely cross-linked one maintains the structural integrity of hydrogels, endowing the DN gels with extraordinary toughness and strength. Haraguchi et al. fabricated a kind of nanocomposite hydrogels (NC gels) using clay nanosheets as multifunctional cross-linking agents [27,28]. It was considered that the uniformly distributed clay nanosheets and their noncovalent interactions (e.g., hydrogen bonding and electrostatic attraction) with polymer chains were responsible for the excellent mechanical properties of the NC gels. The development of these novel hydrogels with unique structures and different energy-dissipation mechanisms greatly enlarged the application range of hydrogel materials.

Self-healing capacity refers to a material’s ability to automatically repair damage and recover to its original structure and properties [29,30]. Because self-healing could significantly prolong the lifetime and improve the safe usage of materials, hydrogels with self-healing ability have gained increasing attention in recent years, and various self-healing hydrogels have been successively reported [31,32,33]. However, despite of their great potential in various applications, most of the currently reported self-healing hydrogels either suffer from weak mechanical properties or need sophisticated polymer design. Therefore, the fabrication of self-healing hydrogels combined with outstanding mechanical performance by a facile approach has been the research focus of material sciences. Generally, the driving forces of the self-healing of hydrogels can be divided into dynamic covalent bonds (e.g., Schiff base linkages, dynamic borate bond, and disulfide bond) and noncovalent physical interactions (e.g., hydrogen bond, co-ordination bond, and hydrophobic interaction) [31]. Among the above-mentioned high-strength hydrogels, NC gels employ noncovalent interactions to cross-link the polymer chains. As a result, these noncovalent interactions could also endow NC gels with a self-healing ability in addition to excellent mechanical properties. This hypothesis has already been successfully verified by several groups, and a series of high-strength and self-healing NC gels have been achieved using different nanomaterials, including clay nanosheets [34], graphene oxide (GO) [35], TiO_2_ [36], and Zr(OH)_4_ nanoparticles [37]. In our previous reports, we developed a new type of NC gels using alumina nanoparticles (Al_2_O_3_ NPs) as cross-linkers [38,39]. The hydrogels were prepared by copolymerization of acrylic acid (AA) and the other polymeric monomer (e.g., *N*,*N*-dimethylacrylamide or *N*-vinyl-2-pyrrolidinone) in the colloid solutions of Al_2_O_3_ NPs. It was considered that the cross-linkage of this kind of hydrogels is contributed to the chelation interactions between Al_2_O_3_ NPs and carboxyl groups on the polymer chains [40]. The resultant hydrogels not only exhibit outstanding mechanical properties, but also have a highly transparent appearance and are swelling-resistant, which make them highly prospective in various applications. Since chelation interactions were similar to those noncovalent interactions that exist in other NC gels, it was hoped that this kind of hydrogel would also have a self-healing capacity, similar to their counterparts cross-linked by above-mentioned inorganic nanomaterials. Nevertheless, only negligible self-healing ability was observed, even when the hydrogels were placed in high temperature (~80 °C) or when the healing time was prolonged (~10 days). Furthermore, in our previous reports, we mainly focused on the formation mechanism and the effect of different precursor compositions on the properties of the resultant hydrogels; potential applications based on the special properties of this new kind of NC gels have not been investigated yet. 

In this article, in order to endow alumina cross-linked NC gels with self-healing capacity and explore their potential applications, a new kind of hydrogel, consisting of copolymer of AA and 2-acrylamido-2-methyl propane sulfonic acid (AMPS) and Al_2_O_3_ NPs, was synthesized. The obtained hydrogels not only exhibited outstanding and comprehensive mechanical properties, but also displayed considerable self-healing capacity due to the choice of AMPS as the comonomer. Furthermore, we demonstrate that this kind of hydrogel could be utilized as long-lasting antifogging material due to its water-absorbing capacity and high transparency at both dry and swollen states. The self-healing mechanism and the effect of the Al_2_O_3_ content on the mechanical, swelling, and self-healing properties of the obtained hydrogels were also investigated. 

## 2. Experimental

### 2.1. Materials

AA (>99%) and AMPS (99%) were purchased from Aladdin Co., Shanghai, China. The 2-hydroxy-4′-(2hydroxyethoxy)-2-methyl-propiophenone (Irgacure 2959) and *N*,*N*′-methylenebisacrylamide (BIS) were provided by Energy Chemical Co., Shanghai, China. Alumina nanoparticles (Al_2_O_3_ NPs) with a particle size of 10–20 nm in the form of transparent colloid solution with the concentration of 10% (*w*/*w* in water) were obtained from Jing Rui New Materials Co., Hangzhou, China. All reagents were used as received without further purification. Deionized (DI) water was used for all the experiments. 

### 2.2. Hydrogel Synthesis 

The poly (AA-*co*-AMPS)/Al_2_O_3_-nanocomposite hydrogels (hereafter termed PAS gels) were synthesized according to the method earlier reported by us [39]. Briefly, different amounts of raw Al_2_O_3_ colloid solutions and extra DI water were added into 20 mL glass bottles to obtain Al_2_O_3_ dispersions with 10 g water and different concentration (2%, 4%, 6%, 8%, and 10%) of Al_2_O_3_ NPs. Then, AA (0.21 g) and AMPS (5.59 g) with a monomer ratio of 1:9 and total monomer concentration of 3 mol/L, as well as 20 mg Irgacure 2959, were added in the above solutions, followed by magnetic stirring for 30 min under a nitrogen atmosphere to result in transparent and homogeneous precursor solutions. Finally, the precursor solutions were injected into different glass molds after being degassed by vacuum, and then exposed to UV (365 nm, 60 mW/cm^2^) irradiation for 30 min to obtain PAS gels. Prior to the further measurements and characterizations, the prepared PAS gels were taken out from the molds and thoroughly washed with running water to remove any residue monomers and impurities on the surface. The obtained hydrogels were denoted as PAS-*x* gels, in which the *x*% represents the Al_2_O_3_ concentration in the initial Al_2_O_3_ dispersions. Furthermore, the hydrogels formed by the neat copolymer of AA and AMPS, as well as the hydrogel cross-linked by organic cross-linker BIS, were prepared for comparison.

### 2.3. Characterizations

The Fourier transform infrared (FT-IR) spectra were obtained using an IRAffinity-1S (Shimadzu Co., Kyoto, Japan) infrared spectrometer with an attenuated total reflectance (ATR) accessory. The UV−Vis transmission spectra were recorded using a UV-1800 (Shimadzu Co., Kyoto, Japan) spectrophotometer using the DI water as the reference. The scanning electron microscopy (SEM) was performed on a Quanta 250 (FEI CO., Ltd., Hillsboro, OR, USA) field emission scanning electron microscope at an acceleration voltage of 20 kV. The atomic force microscopy (AFM) image was taken on a Prima (NT-MDT Co., Moscow, Russia) instrument in tapping mode under an ambient environment. 

### 2.4. Mechanical Tests

The mechanical properties of PAS gels were tested on an AGS-J (Shimadzu Co., Kyoto, Japan) electronic universal testing machine. Sheetlike hydrogel samples with a size of 10 mm × 50 mm × 2 mm were used for the tensile tests. Samples were clamped between two clamps with a gauge length of 10 mm and stretched at the crosshead speed of 100 mm/min until fracture. Fracture stress, elastic modulus, and elongation at break were calculated according to our previous report [39]. The loading–unloading test was carried out using the same instrument at the same tensile speed described above. The samples were firstly stretched to preset strains (400%, 600%, 800%, 1000%, and 1200%), and then unloaded at the same speed to the original gauge length (10 mm) to obtain the loading–unloading profiles. Total energy and dissipated energy were calculated from the area below the loading curves and the area between the loading–unloading profiles, respectively. The dissipation ratio is determined by the dissipated energy divided by the total energy. For the compressive tests, the cylinder-like samples with a diameter of 10 mm and height of 10 mm were compressed to 90% of their original heights at the crosshead speed of 1 mm/min, and compressive strength and compressive modulus were calculated based on the original cross-sectional area of the samples and the slopes between the strain of 10%–20% on the stress–stain curves. To avoid water loss during the tests, a thin layer of silicon oil was coated on the gel specimens. Three measurements were conducted on each hydrogel and the average values were used for discussion. 

### 2.5. Swelling Measurements

The swelling measurements of PAS gels were conducted in DI water at 25 °C. The as-prepared hydrogel samples with a size of 10 mm × 10 mm × 2 mm were firstly dried in an oven at 90 °C, and then immersed in DI water until swelling equilibrium. After a certain time interval, samples were taken out and weighed after removing the water from the surface. The time-dependent swelling ratios (SR) and equilibrium swelling ratios (ESR) of the PAS gels were calculated as follows:

(1) $$\mathrm{SR}=\frac{Wt-Wd}{Wd}\;\mathrm{ESR}=\frac{Ws-Wd}{Wd}$$

In which *W*d, *W*t, and *W*s are the weights of samples at a dry state, a certain time point, and the swelling equilibrium state, respectively. Three measurements were conducted on each hydrogel and the average values were used for discussion.

### 2.6. Self-Healing Experiments

Two rodlike (Φ 5.5 × 60 mm) hydrogel samples with the same composition but different colors were cut into two blocks by a razor blade, and two freshly cut surfaces from different samples were immediately brought into contact. Then, samples were stored in sealed glass bottles to avoid water evaporation and placed at room temperature for different durations (1–24 h). After a preset time, tensile tests were conducted on the healed hydrogels to evaluate their self-healing capacity. The self-healing efficiency of PAS gels is defined by the ratio between the fracture strain of the healed hydrogels and that of the original uncut samples with the same composition, which can be calculated by the following equation:

(2) $$\mathrm{Self}\text{-}\mathrm{healing}\;\mathrm{efficiency}=\frac{St}{So}\times 100\%$$

In which *St* and *So* are the fracture strain of healed samples and that of the original uncut hydrogels, respectively. Three measurements were conducted on each hydrogel, and the average values were used for discussion.

### 2.7. Antifogging Evaluation 

The antifogging evaluation of the PAS gels were conducted on the glass matrix. The hydrogel precursor with the composition of PAS-10 was carefully injected between two glass plates separated by a 1.0 mm thickness rubber spacer. After UV (365 nm, 60 mW/cm^2^) irradiation for 30 min, one of the glass plates was removed and the hydrogel film-coated glass was obtained. The coated glass was placed at room temperature for 24 h and then used for characterizations and antifogging evaluation. 

## 3. Results and Discussion

### 3.1. Formation and Cross-Linking Mechanism of PAS Gels

**Figure 1:**
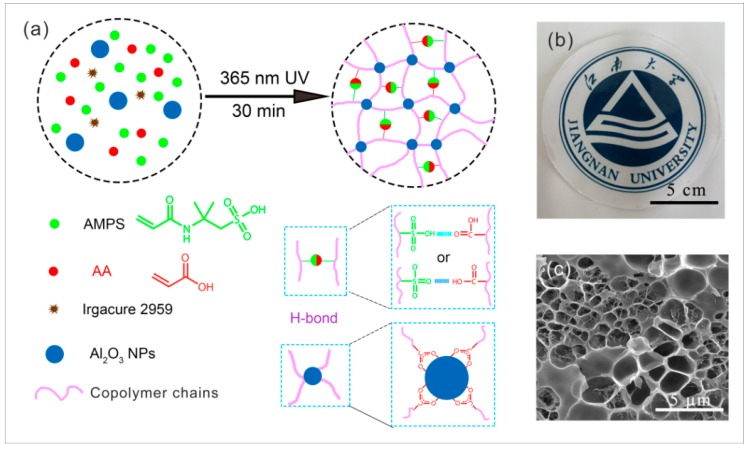
(**a**) Preparation and cross-linking mechanism of poly (AA-*co*-AMPS)/Al_2_O_3_-nanocomposite hydrogels (PAS gels); (**b**) digital picture of as-prepared PAS gels; (**c**) typical scanning electron microscopy (SEM) image of PAS-6 gel.

**Figure 2:**
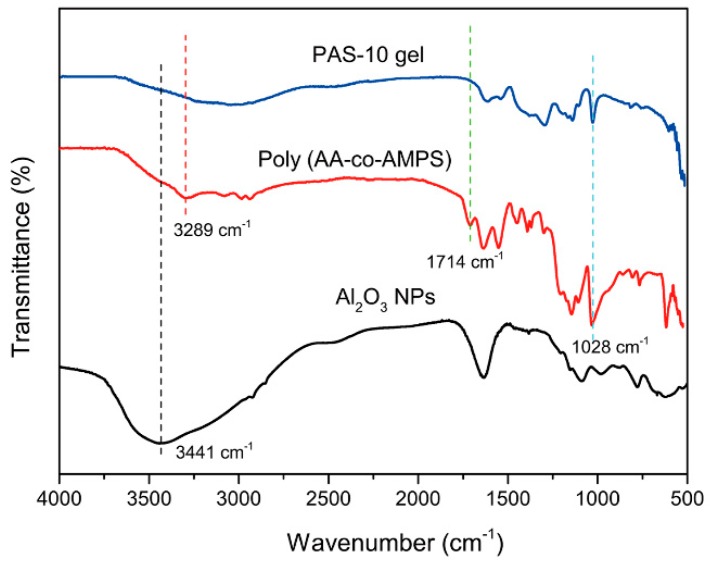
Fourier transform infrared (FT-IR) spectra of Al_2_O_3_ NPs, neat copolymer of acrylic acid (AA) and 2-acrylamido-2-methylpropane sulfonic acid (AMPS), and PAS-10 gel.

The PAS gels were synthesized by facile UV-initiated in situ free radical polymerization as illustrated in Figure 1a. Homogeneous precursor solutions were firstly obtained by mixing all the components, including the Al_2_O_3_ colloid solution, water, monomers, and photoinitiator, under a nitrogen atmosphere. Upon UV irradiation, the photoinitiator breaks down to generate free radicals, which initiate the copolymerization of AA and AMPS. Simultaneously, the formed polymer chains interact with the Al_2_O_3_ NPs to form cross-linked three-dimensional (3D) network structures. The resultant hydrogels were highly transparent (Figure 1b), and displayed interconnected porous microstructures (Figure 1c and Figure S1) similar to NC gels cross-linked by other nanomaterials [37,41,42,43]. To investigate the interactions contributing to the formation of PAS gels, FT-IR spectroscopy was conducted on the Al_2_O_3_ NPs, neat copolymer of AA and AMPS, and the PAS gels. As shown in Figure 2, the absorption peak at 3441 cm^−1^ in the spectrum of the Al_2_O_3_ NPs and the band centered at 1714 cm^−1^ in the spectrum of the neat copolymer disappeared in the spectrum of the PAS gels. According to the previous reports [39,40], this is because of the formation of chelation reactions between –COOH groups on polymer chains and Al_2_O_3_ NPs. Furthermore, since the Al_2_O_3_ NPs in the colloid solution are positively charged, the electrostatic interactions between the Al_2_O_3_ NPs and –COO^−^ groups may be beneficial to the formation of chelation reactions within PAS gels. In addition, it can also be seen that the characteristic peak of –OH and S=O groups on polymer chains shifted to 3289 and 1028 cm^−1^, respectively, in the spectrum of the neat copolymers, which are lower to their normal positions (~3400 cm^−1^ for the –OH groups and 1038 cm^−1^ for the S=O groups). This implies the existence of hydrogen bonds between polymer chains as illustrated in Figure 1a. This conclusion has been verified by the fact that the neat copolymer of AA and AMPS could form solidlike materials (without Al_2_O_3_ NPs) with an appearance similar to the PAS gels. However, the formed materials would be dissolved within two days when immersed in water (Figure S2), indicating that the hydrogen bonds are not strong enough to form stable cross-linkage within polymer networks. In contrast, after Al_2_O_3_ NPs were introduced, the obtained hydrogels were highly stable, which could keep their structural integrity even after being placed in water for 30 days. Based on the above analysis, it is reasonable to conclude that the chelation reactions between –COOH groups on polymer chains and Al_2_O_3_ NPs were responsible for the cross-linking structures of PAS gels, and the hydrogen bonds between polymer chains may also contribute to their network structures.

### 3.2. Mechanical and Swelling Properties of PAS Gels

**Figure 3:**
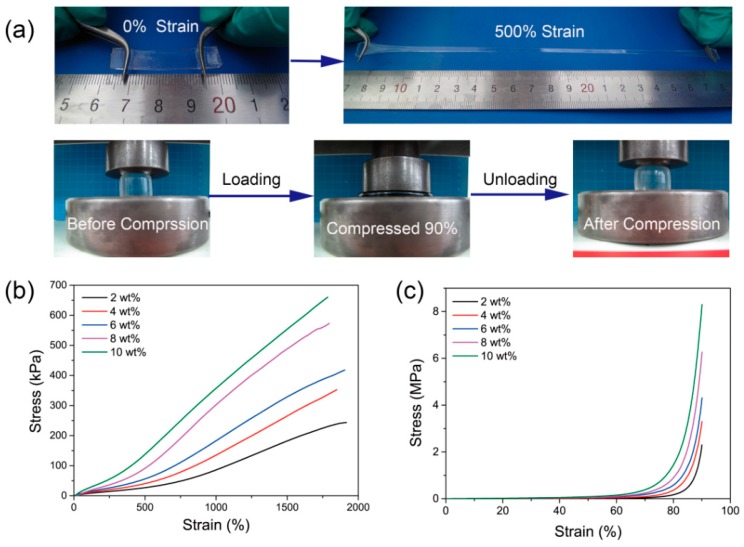
(**a**) Photo illustration of the mechanical properties of PAS gels; (**b**) typical tensile and (**c**) compressive stress–strain curves of PAS gels with different Al_2_O_3_ content.

Based on the chelation reactions between Al_2_O_3_ NPs and the polymer matrix, the PAS gels exhibited excellent mechanical properties. As depicted in Figure 3a, the PAS gels could be stretched to five times their original length by hand using two tweezers, revealing outstanding extensibility and damage tolerance. Furthermore, the PAS gels were able to keep intact under large-scale compression (90% of their original heights), and recover to their original dimensions with negligible residue strain upon unloading within 10 min, showing good toughness and self-recoverability. In contrast, the hydrogel cross-linked by BIS is brittle and fragile, which could be easily fractured by small external force (Figure S3a). Although the hydrogel formed by the neat copolymer of AA and AMPS reveals excellent toughness (Figure S3b), it would quickly dissolve in water (Figure S2), making it useless in practical applications. These results indicate that Al_2_O_3_ NPs play a vital role in the mechanical properties of PAS gels. To systematically study the mechanical properties of PAS gels, tensile and compressive tests were conducted. Because PAS gels share the same cross-linking mechanism as that described in our previous report [39], it was found that the effect of AA to AMPS ratios on the properties of PAS gels were also similar to that previously reported. That is, increasing the AA ratio would lead to an increase in the cross-linking density of PAS gels. Thus, elongation at the break as well as swelling ratio consequently decrease, while the elastic modulus of PAS gels increases. Therefore, in this study, we focused on how the amount of Al_2_O_3_ NPs influences the properties of PAS gels. Figure 3c shows the tensile stress–strain curves of PAS gels with different Al_2_O_3_ content, and elongation at break, tensile strength, and elastic modulus are listed in Table 1. It can be seen that PAS gels could be stretched nearly 20-fold their original length before fracture, with tensile strength ranging from 240 to 660 kPa, depending on their Al_2_O_3_ content. Furthermore, it was found that both the elastic moduli and tensile strength of the PAS gels increased as Al_2_O_3_ content increased, while the elongations at break were almost unchanged. This result is quite similar to classical NC gels cross-linked by clay nanosheets [44,45], confirming that the Al_2_O_3_ NPs act as inorganic cross-linking agents in PAS gels. Figure 3c displays the compressive stress–strain curves of PAS gels. It reveals that all PAS gels were able to withstand 90% compression without breaking, showing compressive strength up to 8.30 MPa. The detailed data of the compressive tests are also summarized in Table 1. Similar to the tensile tests, the elastic moduli and compressive strength also increased as Al_2_O_3_ content increased. Based on the above results, it is worth noting that the mechanical properties of PAS gels could be readily tuned by altering the Al_2_O_3_ content in the initial precursor solutions. Although the highest tensile and compressive strength of PAS gels in the current study were only 660 kPa and 8.30 MPa respectively, PAS gels with higher mechanical strength could be obtained by further increasing the Al_2_O_3_ content within the polymer matrix.

**Figure 4:**
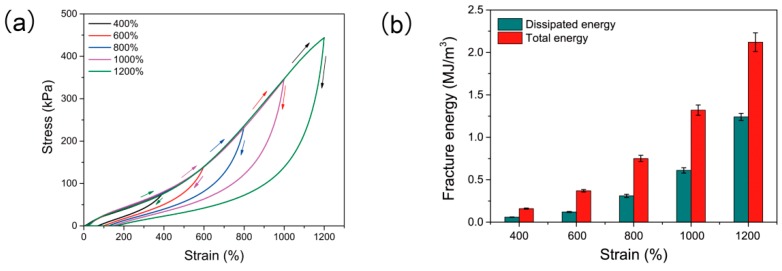
(**a**) Loading–unloading profiles of PAS gels with different strains; (**b**) total fracture energy and dissipated fracture energy calculated from (**a**).

To further assess the toughness of PAS gels, the cyclic tensile loading–unloading tests were performed on the PAS-10 gel. As depicted in Figure 4a, pronounced hysteresis loops were observed for all the tests, indicating that the PAS gels could effectively dissipate applied energy [41,46]. This result also verifies the non-covalent cross-linking structures of PAS gels. It has been discussed above that there exists hydrogen bonds and chelation reactions within PAS gels. Upon loading, part of the hydrogen bonds and chelation reactions acted as sacrificial bonds that broke to dissipate energy, while the rest of them maintained the macroscopic integrity of PAS gels. During the unloading process, the broken hydrogen bonds and chelation reactions reformed to regain the configuration of PAS gels. However, unloading time was too short to allow all the broken bonds to completely reform, leading to the large hysteresis loops. The quantified data calculated from the loading–unloading profiles are shown in Figure 4b. It can be seen that, with the increase in strain, more energy could be dissipated and higher energy-dissipation ratios were observed. For example, when the loading strain was 400%, the total energy and dissipated energy were 0.16 and 0.06 MJ/m^3^, respectively, and the dissipation ratio was calculated to be 37.5%. As strain increased from 600% to 1200%, the dissipation ratio also changed from 39.3% to 56.1%. This phenomenon further proves the excellent energy-dissipation ability of PAS gels, which contributes to their outstanding mechanical performance. 

**Figure 5:**
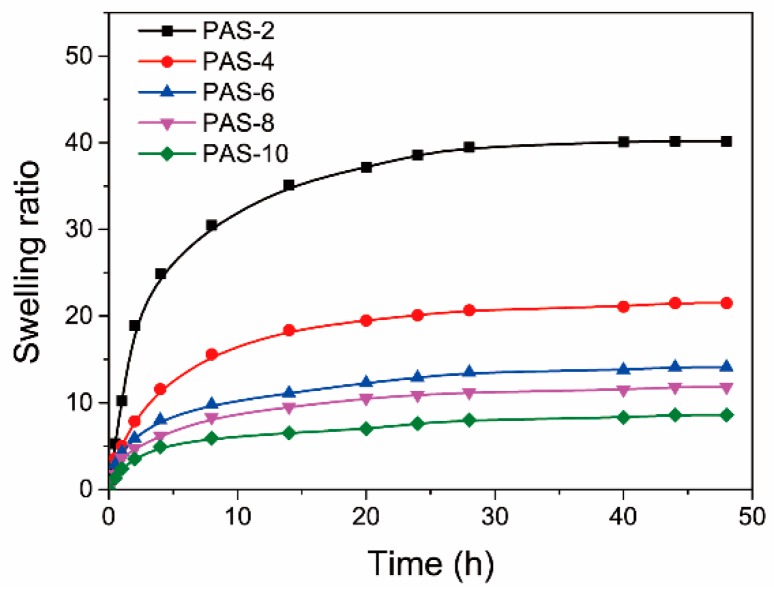
Time-dependent swelling ratios of PAS gels.

The water-absorbing capacity of hydrogels is an important factor for their practical applications. Therefore, the swelling ratios of PAS gels were measured in DI water at 25 °C. Figure 5 shows the time-dependent swelling ratios of PAS gels. It reveals that the PAS gels could reach swelling equilibrium from dry state within 48 h, and show ESR ranging from 8.5 to 39.5, indicating fast and excellent water-absorbing capacity. In addition, both the swelling rate and ESR monotonously decreased as Al_2_O_3_ content increased, which also proves that Al_2_O_3_ NPs act as cross-linking agents within PAS gels. Here, it should be noted that both the dried and swollen hydrogels displayed transparent appearances as their as-prepared counterparts, endowing the PAS gels with potential applications in optical devices.

### 3.3. Self-Healing Ability of PAS Gels

**Figure 6:**
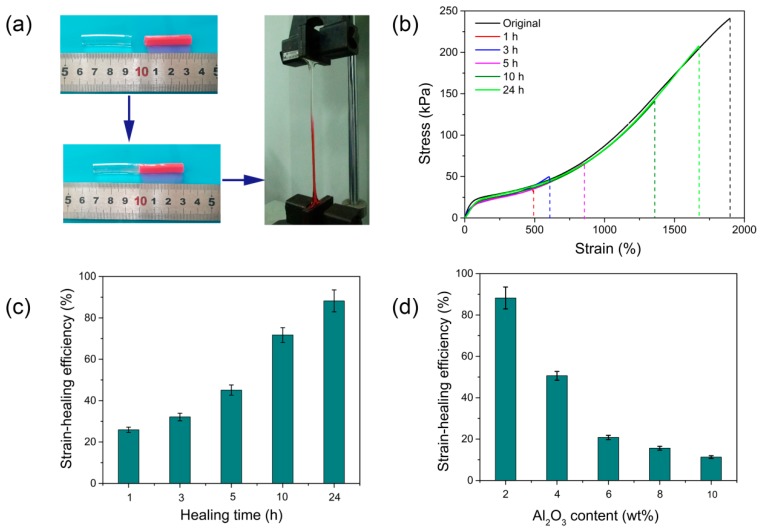
(**a**) Illustration of self-healing capacity of PAS gels; (**b**) stress–strain curves of the original and healed PAS-1 gel; (**c**) self-healing efficiency of PAS-2 gel after healing for different time; (**d**) self-healing efficiency of PAS gels with different Al_2_O_3_ content after healing for 24 h.

On the basis of the noncovalent cross-linking mechanism, as well as the existence of strong hydrogen bonds within polymer chains, PAS gels also revealed remarkable self-healing capacity similar to other NC gels [34,35,37]. As shown in Figure 6a, two pieces of hydrogel samples were put together immediately after being cut. Upon being sealed and stored in a glass bottle for 24 h, the surfaces of the two samples were merged together, and the healed sample could be stretched to a certain strain without fractures, which confirms the self-healing ability of PAS gels. To quantitatively evaluate the self-healing capacity of PAS gels, tensile tests were performed on the original and healed PAS-2 gel. The stress–strain curves and self-healing efficiency based on fracture strains are displayed in Figure 6b,c, respectively. It was found that the self-healing performance of PAS-2 gel could be significantly enhanced by prolonging the healing time. When the healing time was 1 h, the healed sample displayed elongation at break and tensile strength with 490.6% and 34.8 kPa, respectively. Self-healing efficiency based on the fracture stain of original PAS-2 gel was only 25.9%. As healing time increased, the fracture stress and strain were both significantly enhanced. After healing for 24 h, self-healing efficiency reached 88.2%, indicating excellent self-healing capacity. It is widely accepted that the self-healing property of NC gels is attributed to the diffusion of polymer chains across the hydrogel interface followed by the reformation of noncovalent interactions at the interface [31,34]. As for the PAS gels, when the two surfaces were closely contacted, the poly(AA-*co*-AMPS) chains at the two surfaces diffused into each other and interacted with the Al_2_O_3_ NPs and other polymer chains via chelation reactions or hydrogen bonds, respectively. Consequently, new cross-links are formed at the interface, leading to the adherence of the cut surfaces. Prolonging healing time would allow more polymer chains to diffuse across the interface and form more interactions, thus resulting in higher self-healing efficiency. Here, it should be noted that the self-healing efficiency of the hydrogels cannot achieve 100% even after much longer healing time due to the irreversible fracture of the polymer chains. 

In addition to healing time, it was found that the self-healing efficiency of PAS gels is also influenced by the Al_2_O_3_ content within hydrogels, as shown in Figure S4 and Figure 6d. The PAS-2 gel showed self-healing efficiency of nearly 90% after healing for 24 h. However, as the content of Al_2_O_3_ increased to 4%, self-healing efficiency sharply decreased to 50.6% after the same healing time. Further increasing the Al_2_O_3_ content leads to the self-healing efficiency drop to 20.8% (PAS-6 gel), 15.6% (PAS-8 gel), and 11.3% (PAS-10 gel). This is because that the increase of Al_2_O_3_ content leads to an increase of cross-linking density of the PAS gels, which causes a decrease in the length of the polymer chains between the neighboring cross-linking points. Compared with longer polymer chains, shorter chains were more difficult to diffuse across the interface [34]. As a result, the self-healing efficiency of PAS gels significantly decreased. 

### 3.4. Antifogging Application of PAS Gels 

**Figure 7:**
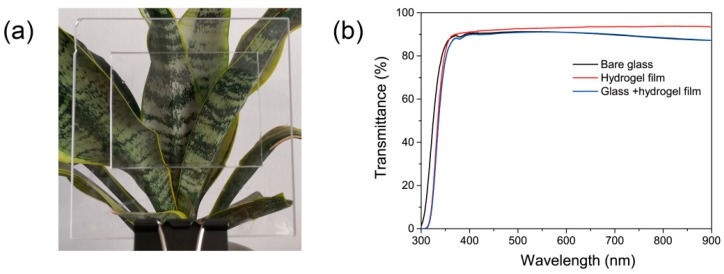
(**a**) Digital photograph of the PAS gel-coated glass plate; (**b**) UV–Vis transmission spectra of bare glass, hydrogel film, and the hydrogel-coated glass plate.

In previous reports, NC gels were investigated for various applications, such as tissue engineering [47], water treatment [36], strain sensors [48], and 3D printing [49] due to their unique physiochemical properties. Herein, based on the water-absorbing ability and the highly transparent appearances of PAS gels at both dry and swollen states, we attempted to explore the applications of PAS gels as potential antifogging materials. For this purpose, the PAS-10 gel film was directly prepared on glass plates with the method described in Section 2.7. Figure 7a displays the digital photograph of a hydrogel-coated glass plate in front of a green plant. It can be seen that both of the uncoated and coated areas were highly transparent, indicating that the coating of the PAS-10 gel on the glass matrix would not affect glass transparency. In fact, the PAS-10 gel film displayed higher transparency than that of the used glass plate, as shown in Figure 7b, which means that the transparence of the coated glass is determined by the glass matrix instead of the hydrogel film. In addition, our experimental results also revealed that the thickness and the Al_2_O_3_ content of the PAS gels would not influence the transparency of the hydrogel-coated glass.

**Figure 8:**
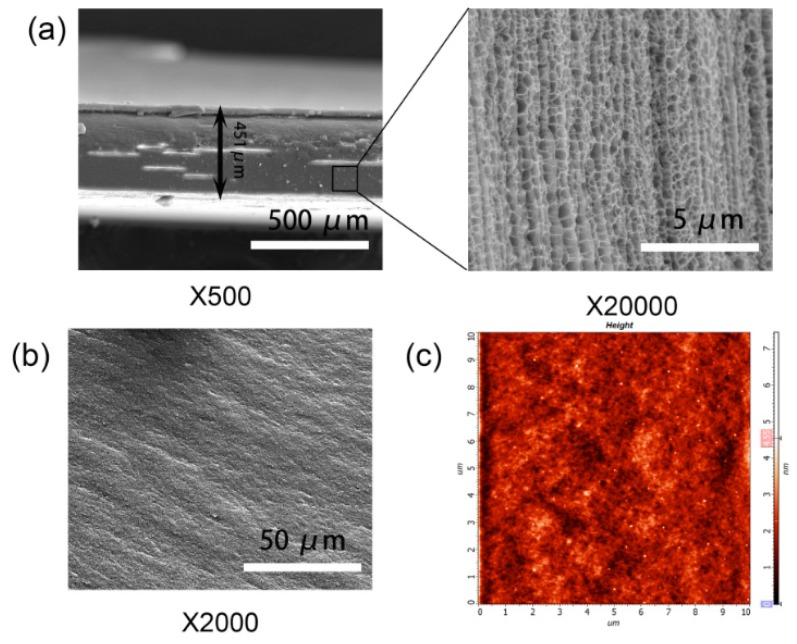
(**a**) Typical SEM images of cross-section of PAS gel coating with different magnifications; (**b**) SEM image of the surface of PAS gel coating; (**c**) atomic force microscopy (AFM) image of the surface of PAS gel coating.

In order to characterize the morphology of the PAS gel coating on glass plate, SEM and AFM were performed, and the typical images are shown in Figure 8. Figure 8a displays the SEM images of the cross-section of PAS-10 gel coating under different magnification. The thickness of the as-prepared PAS-10 gel coating was around 1.0 mm, determined by the used mold. After being placed at room temperature for 24 h, the gel coating lost most of the water within the hydrogels, and thickness was reduced to around 450 μm. Interestingly, it was found that the dried gel coating revealed a well-ordered porous structure, although the pore size was much smaller that of the as-prepared PAS gels after freeze drying. This phenomenon implies that the 3D network structure of hydrogel was not totally destroyed during the drying process. In addition, the existence of this porous structure may be beneficial to its fast water absorbing ability. Apart from the cross-section, we also observed the surface morphology of the gel coating. Both of the SEM and AFM images shown in Figure 8c,d verified the excellent smoothness of the surface of the gel coating, which could prevent the scattering of the light and endow the gel-coated glass plate with high transparency. 

**Figure 9:**
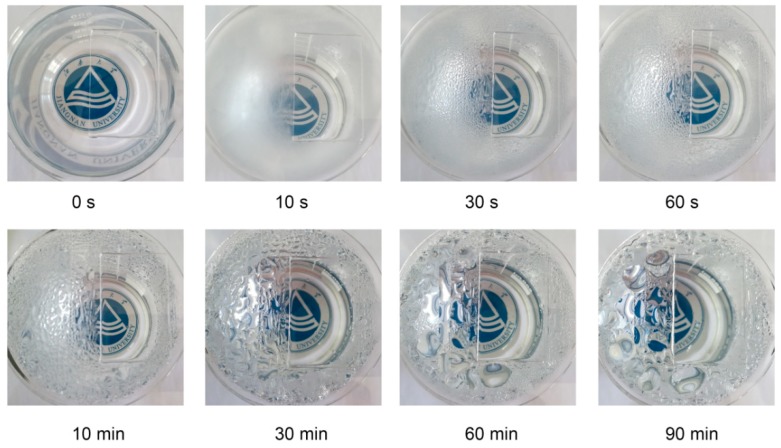
Illustration of the antifogging property of the PAS gels.

To illustrate the antifogging performance of the PAS gels, the hydrogel-coated glass was placed above 95 °C hot water and the antifogging property of hydrogels was recorded with a digital camera as shown in Figure 9. Before measurement, the partially coated glass plate was placed on top of an empty beaker. It can be seen that both the coated and uncoated areas were highly transparent, and the logo of Jiangnan University under the beaker can be clearly viewed. When the beaker was filled with hot water, the uncoated area immediately (within 10 s) became blurred due to the formation of fog on the surface. In contrast, the coated area still stayed highly transparent due to the fast absorption of the vapor into the hydrogel coating, preventing the formation of small water droplets on the surface. As testing time went on, some small water droplets formed on the uncoated area, starting to merge into big drops, while the coated area was still transparent even after testing for 90 min, displaying a long-lasting antifog property. Furthermore, it is worthy to note that the PAS gel film was firmly stuck on the glass plate throughout the whole testing process, and no buckling or detachment of hydrogel film was observed. The reasons for this phenomenon may be explained as follows. Firstly, the hydrogel film was prepared directly on the glass plate from the hydrogel precursor. The aqueous precursor could diffuse to any defective parts on the glass plate, forming strong hydrogen bonding and Van der Waals force with the glass plate after polymerization. Therefore, it could still firmly stick on the glass plate even as the hydrogel film was filled with some water. Secondly, as shown in the Figure 8b, the dried hydrogel film coated on the glass still possessed a porous structure that could accommodate a large number of water molecules without forming buckling. Although some polymer-related antifogging materials have been documented in the literature [50,51,52,53], to the best of our knowledge, this is the first report on the utilization of NC gels for antifogging applications. Based on the excellent antifogging property and facile preparation of PAS gels, it is expected that this novel hydrogel could be utilized as antifogging material on car windshields, goggles, and various optical devices to ensure the transparency of their surfaces in humid environments.

## 4. Conclusions

In summary, we synthesized a polymer/Al_2_O_3_ nanocomposite hydrogel with excellent mechanical properties, outstanding self-healing capacity, and a long-lasting antifogging property. The hydrogels were prepared via facile in situ free radical polymerization of AA and AMPS using Al_2_O_3_ NPs as cross-linker agents. It was revealed that the noncovalent chelation reactions between Al_2_O_3_ NPs and the polymer matrix, as well as the hydrogen bonds between polymer chains, contribute to the high mechanical strength and self-healing capacity. Interestingly, the hydrogels displayed long-lasting antifogging property when coated on a glass matrix due to their water-absorbing ability as well as their high transparency at both dry and swollen states. We believe that this new type of nanocomposite hydrogel would find applications in various hydrogel-based devices, including soft robots, artificial muscles, and optical devices.

## Figures and Tables

**Table 1:** Mechanical properties of PAS gels.

Al_2_O_3_ Content	Tensile Strength/kP	Elongation at Break/%	Tensile Modulus/kPa	Compressive Strength/MPa	Compressive Modulus/kPa
**2%**	243.5 ± 12.3	1916.3 ± 105.5	4.6 ± 0.21	2.29 ± 0.11	20.4 ± 1.03
**4%**	352.3 ± 15.8	1848.5 ± 103.7	6.7 ± 0.35	3.30 ± 0.15	36.6 ± 1.98
**6%**	417.9 ± 24.3	1904.5 ± 104.7	8.6 ± 0.41	4.31 ± 0.18	65.3 ± 3.30
**8%**	572.5 ± 27.9	1793.8 ± 90.6	14.1 ± 0.78	6.27 ± 0.32	78.5 ± 3.62
**10%**	659.3 ± 30.6	1784.9 ± 92.5	20.1 ± 1.02	8.30 ± 0.42	110.1 ± 5.51

## Supplementary Materials

The following are available online at http://www.mdpi.com/2073-4360/10/12/1362/s1. Figure S1. SEM images of PAS gels with different Al_2_O_3_ content. Figure S2. Illustration of the dissolution of neat poly(AA-*co*-AMPS) gels without Al_2_O_3_ NPs. Figure S3. Illustration of mechanical properties of BIS cross-linked hydrogel and neat poly(AA-*co*-AMPS) hydrogel. Figure S4. Stress–strain curves of original and healed PAS gels.
