# Supplementary material for: Synthesis, Characterization, and Antifogging Application of Polymer/Al2O3 Nanocomposite Hydrogels with High Strength and Self-Healing Capacity
Source: Polymers (Basel). 2018 Dec 8;10(12):1362. doi: 10.3390/polym10121362 (PMC6401749; doi:10.3390/polym10121362)
Supplement: Supplementary file 1 [file polymers-10-01362-s001.pdf]

Supplementary Information for

Synthesis, Characterization and Anti-fogging Application of Polymer/Al_2O_3 Nanocomposite Hydrogels with High Strength and Self-healing Capacity

Bo Xu, Yuwei Liu, Jiugang Yuan, Ping Wang, and Qiang Wang *

Key Laboratory of Eco-Textile, Ministry of Education, College of Textile and Clothing, Jiangnan University, Wuxi 214122, P. R. China.

* Correspondence: qiang_wang@163.com; Tel.: +86-510-8591-2005

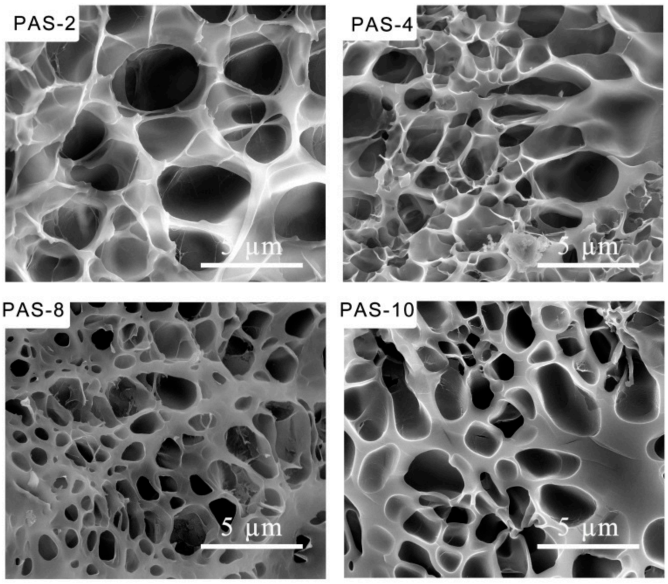

Figure S1. SEM images of PAS-2 gel, PAS-4 gel, PAS-8 gel and PAS-10 gel.

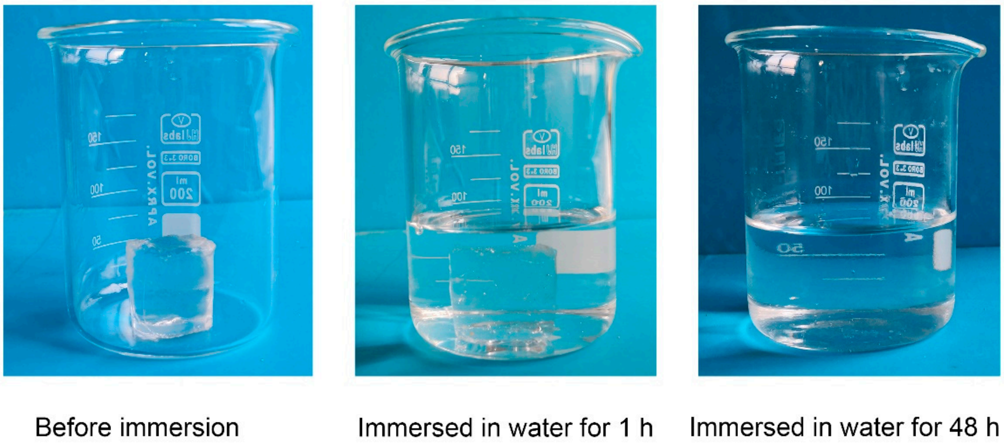

Figure S2. Illustration of the dissolution of neat poly (AA-co-AMPS) gels without Al_2O_3 NPs

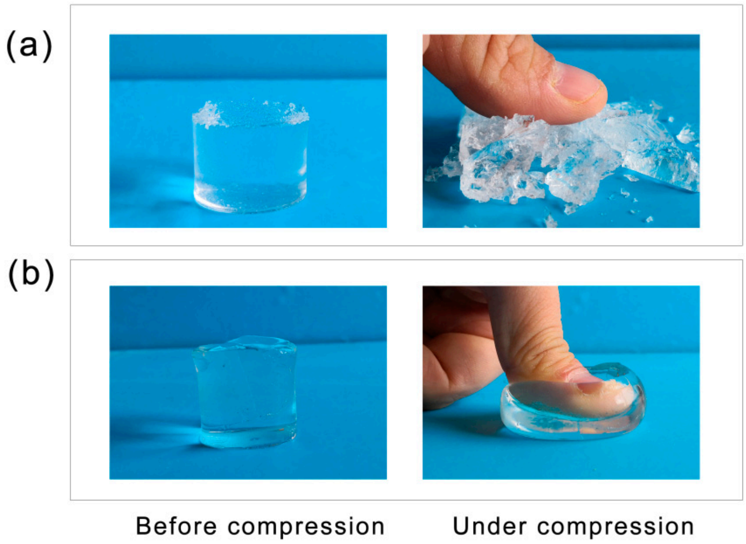

Figure S3. Illustration of mechanical properties of (a) BIS cross-linked hydrogel and (b) neat poly (AA-co-AMPS) hydrogel.

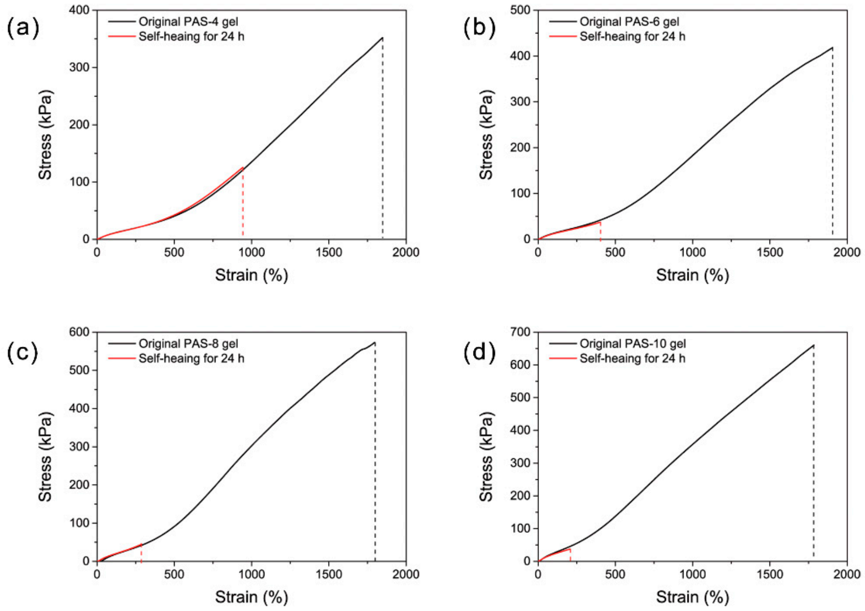

Figure S4. Stress-strain curves of original and healed PAS gels
